# Supplementary material for: Predicting in-Hospital Mortality of Patients with COVID-19 Using Machine Learning Techniques
Source: J Pers Med. 2021 Apr 24;11(5):343. doi: 10.3390/jpm11050343 (PMC8147079; doi:10.3390/jpm11050343)
Supplement: Supplementary file 1 [file jpm-11-00343-s001.zip › jpm-1157462-supplementary.pdf]

*Table S1***.** Differences in ROC (Panel A) and F statistic (Panel B) among the MLTs employed to predict in-hospital death. The plots report the confidence intervals for the difference in performances across resamples. The values below the diagonal of the tables represent the p-values of performance comparison across MLTs, while the numbers above the diagonal are the average differences in performances across metrics.

Panel A

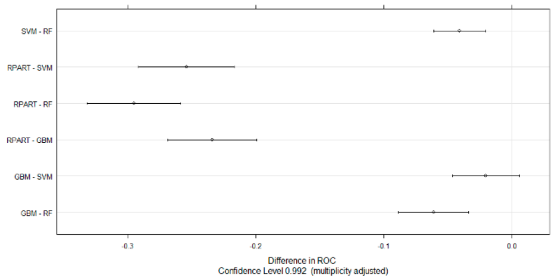

	RPART	GBM	SVM	RF
**RPART**		-0.234	-0.254	-0.295
**GBM**	<0.001		-0.020	-0.061
**SVM**	<0.001	0.2066		-0.041
**RF**	<0.001	<0.001	<0.001	

Panel B

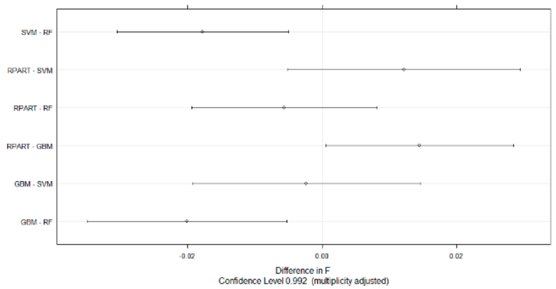

	RPART	GBM	SVM	RF
**RPART**		0.014	0.012	-0.006
**GBM**	0.038		-0.002	-0.020
**SVM**	0.329	0.990		-0.018
**RF**	0.990	0.004	0.003	

Table S2. Variable importance plots for each Random Forest developed.

N= 40 until 13/03/2020

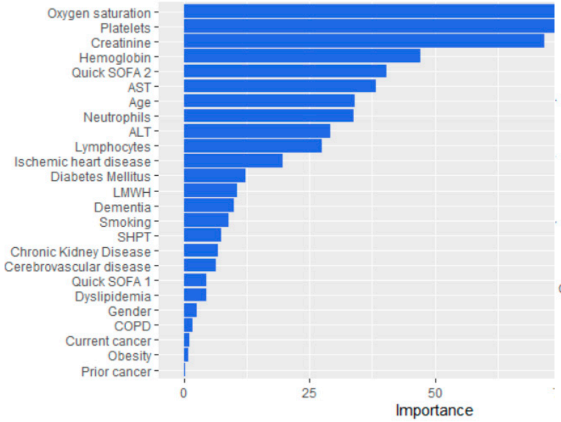

N=60 until 15/03/2020

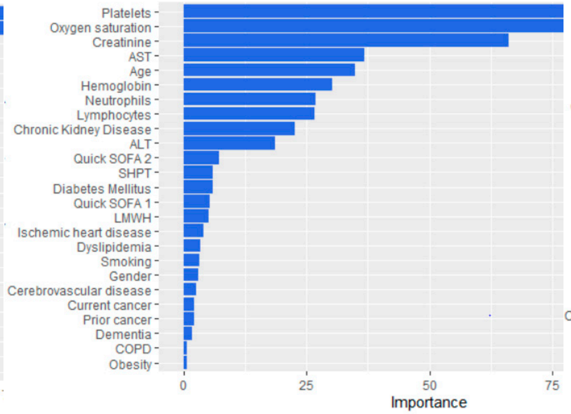

N= 80 until 17/03/2020

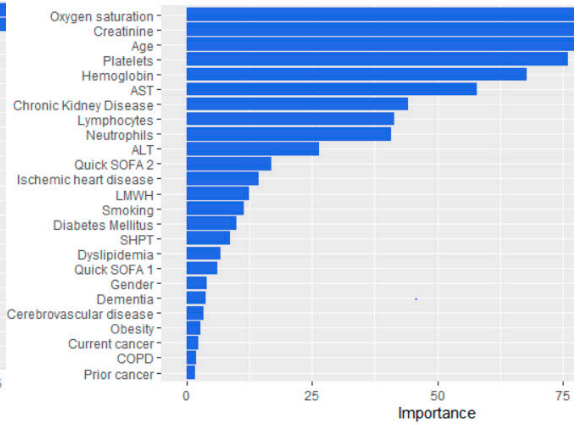

N=100 until 18/03/2020

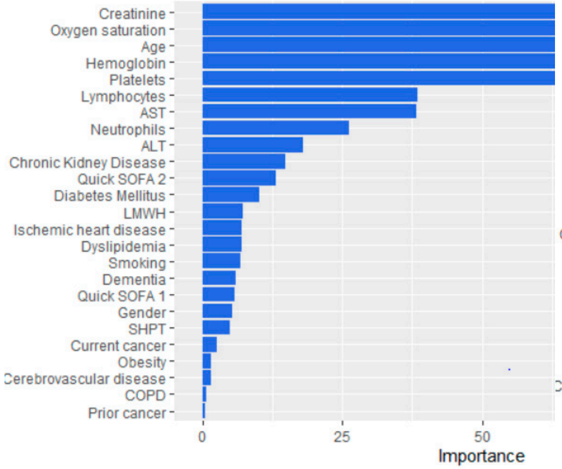

N=120 until 20/03/2020

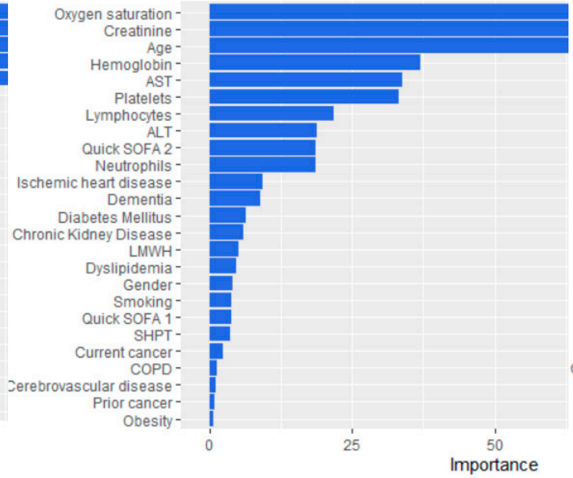

N=140 until 21/03/2020

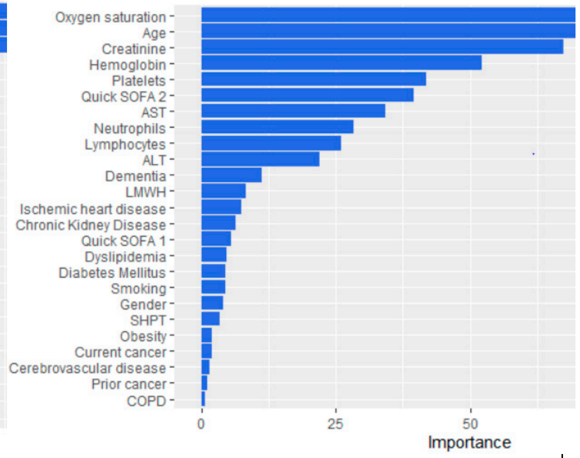

N=160 until 23/03/2020	N=180 until 25/03/2020	N=200 until 27/03/2020

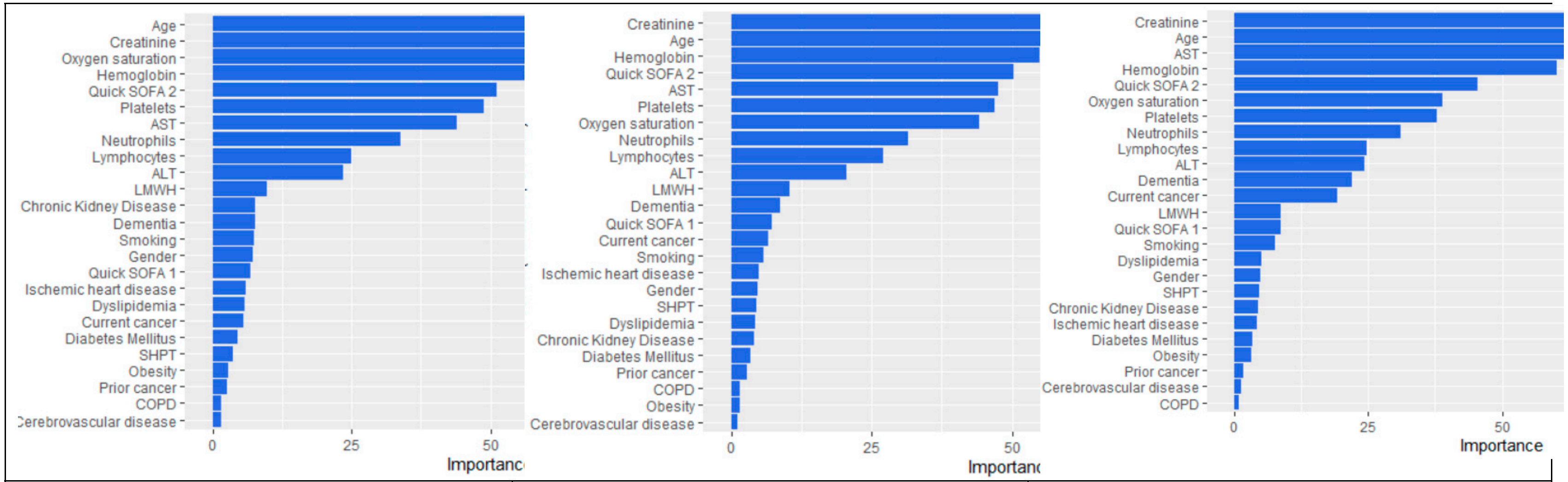

N=220 until 28/03/2020 N=240 until 30/03/2020 N=260 until 02/04/2020

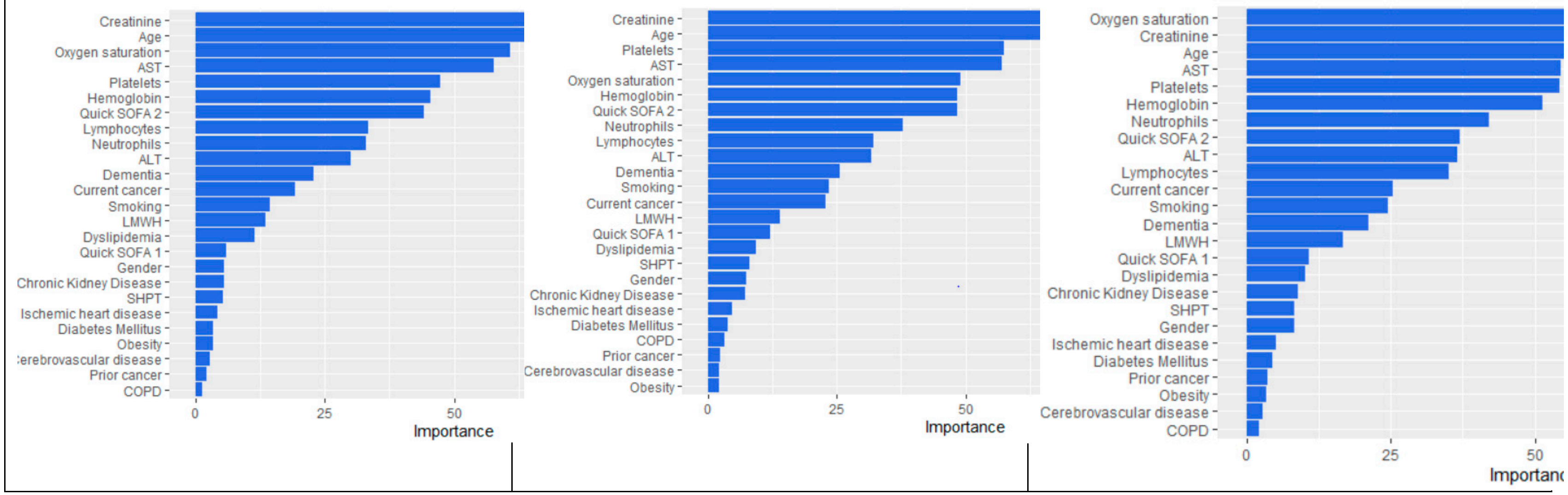

Figure S1. To assess if class imbalance affected the results of the model with the best performance, i.e., the random forest, we tested the hypothesis of difference between the random forest and a null model. The null model represents the typical behavior of an MLT that does not properly handle unbalanced classes leading to biased predictions towards the majority class. The values of ROC, sensitivity, and specificity within the resampling procedures were reported for both the null model and the random forest. The difference between such measures was then tested using the approach proposed by Hothorn et al. [1] and Eugster et al. [2]. Significant differences between the null model and the random forest tool ($P<0.001$) were identified for the three measures of performance, demonstrating the random forest ability to deal with class imbalance.

The figure reports the ROC, sensitivity, and specificity measures of the null model and the random forest.

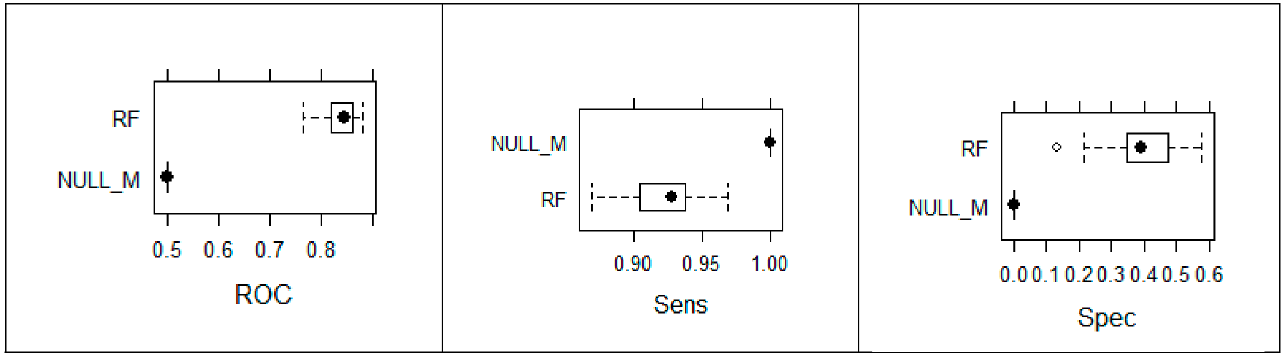

1. Hothorn, T.; Leisch, F.; Zeileis, A.; Hornik, K. The Design and Analysis of Benchmark Experiments. Journal of Computational and Graphical Statistics 2005, 14, 675–699.
2. Eugster, M.J.; Leisch, F. Exploratory Analysis of Benchmark Experiments an Interactive Approach. Computational Statistics 2011, 26, 699–710.
